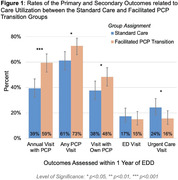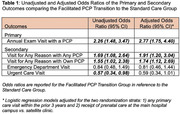# Oral Concurrent Session 9 – Medical Complications: Saturday, February 1, 2025 8:00 AM – 10:30 AM

**DOI:** 10.1002/pmf2.12022

**Published:** 2025-01-05

## 95 | Prevention Of Methamphetamine Use among Postpartum People (PROMPT): A Pilot Randomized Controlled Trial

Marcela C. Smid^1^; Jasmin E. Charles^1^; Amanda A. Allshouse^2^; Stephanie Castro^1^; Grace Humiston^1^; Elysha Cash^2^; Adam G. Gordon^2^; Kristi Carlston^1^; Marie Gibson^1^; Gerald Cochran^1^

*^1^University of Utah Health, Salt Lake City, UT; ^2^University of Utah, Salt Lake City, UT*

8:00 AM ‐ 8:15 AM

**Objective**: In the US, methamphetamine use disorder (MUD) is a leading contributor to postpartum deaths. Our objective was to determine feasibility, safety, and preliminary estimate of efficacy of oral micronized progesterone, a neurosteroid with effects on reward‐reinforcement brain circuitry, to reduce postpartum methamphetamine use (MU).

**Study Design**: We conducted a double‐blind, randomized controlled trial of people with MUD within 12 weeks postpartum comparing oral micronized progesterone to placebo. We excluded those with MU within 4 weeks of enrollment, severe depression/anxiety symptoms or suicidality at screening, taking sedating medications, or < 2 weeks medication for opioid use disorder (MOUD). Block randomization occurred 1:1 stratified for opioid use disorder (OUD). Weekly study visits assessed MU, defined as self‐reported MU or positive urine toxicology, and MU craving. The primary outcome was feasibility, defined as enrollment of 80% planned sample (n = 32/40). Safety was assessed by number of adverse events (AEs) between treatment arms. Preliminary estimate of efficacy was determined by MU and craving scores. Logistic regression accounting for time was used to assess change in craving scores by arms, adjusting for confounders.

**Results**: From November 2021 to January 2024, 253 individuals were screened and 34/46 eligible individuals enrolled and randomized (74%) with 88% retention. At baseline, demographics and craving scores were similar between arms (Table 1). AEs did not differ by arm. For MU, there was no difference between groups by intent to treat (11% vs 0%, p = 0.49) or as treated (6% vs 6%, p = 0.99). Craving reduced for both arms, with a larger, but not significant, decrease in progesterone group (Table 2, Model 1). There was a significant interaction by OUD or MOUD, with progesterone increasing cravings for those without OUD and with OUD taking buprenorphine but reducing cravings for those with OUD taking methadone (Models 5, 6).

**Conclusion**: Promising results of this feasibility and safety trial inform the design of a multi‐center trial of progesterone for prevention of postpartum MU.

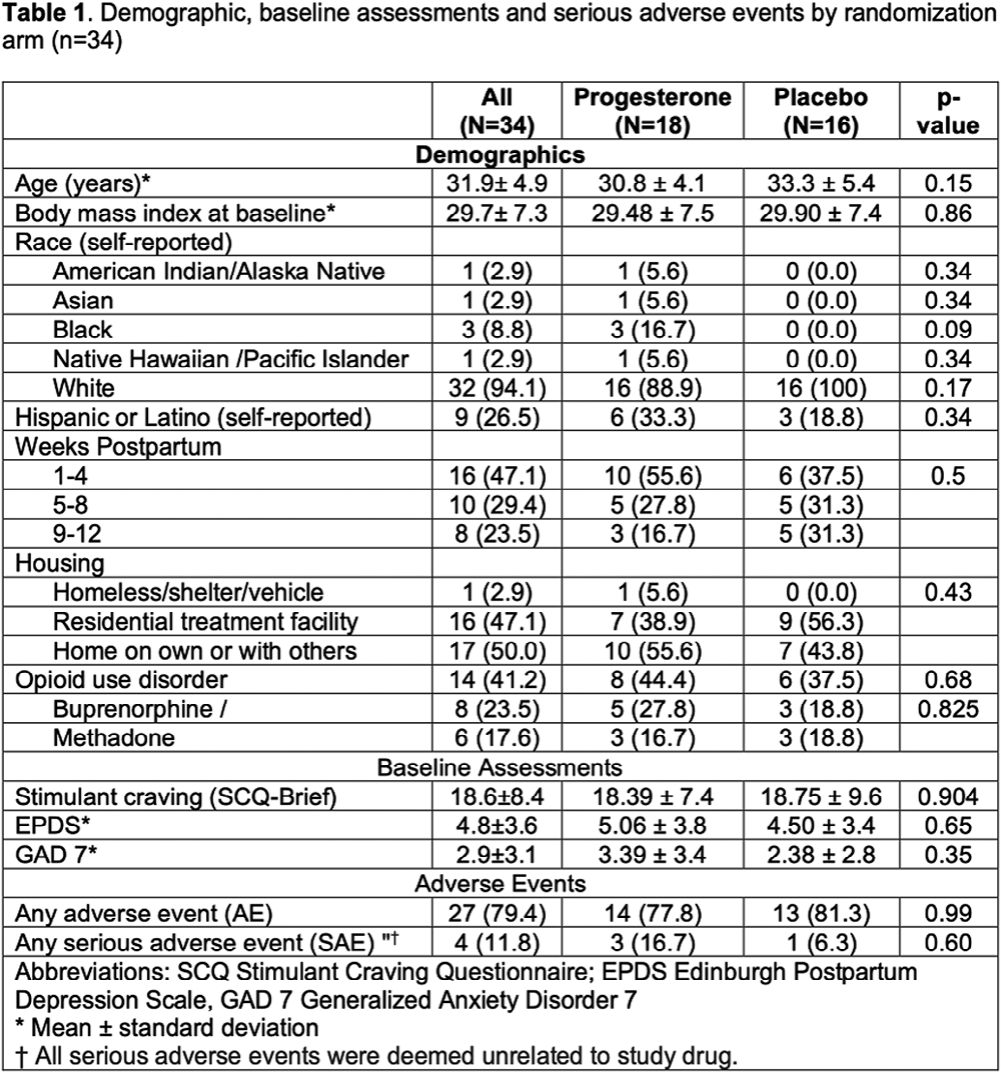

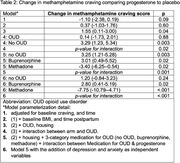

## 96 | Postpartum VTE risk by CHA2DS2‐VASc Score in Patients with Atrial Fibrillation or Flutter During Pregnancy

Virginia Y. Watkins^1^; Miriam L. Estin^2^; Sarah C. Snow^1^; Cary C. Ward^1^; Marie‐Louise Meng^1^; Jerome J. Federspiel^1^

*^1^Duke University School of Medicine, Durham, NC; ^2^Duke university School of Medicine, Durham, NC*

8:15 AM ‐ 8:30 AM

**Objective**: In nonpregnant individuals with atrial fibrillation (AF) or flutter (AFL), the CHA_2_DS_2_‐VASc score guides candidacy for anticoagulation (AC), with AC commonly recommended for a score >2. Whether the CHA_2_DS_2_‐VASc score can guide AC candidacy in pregnant populations with AF/AFL is unclear. In this study, we determined whether rates of postpartum venous thromboembolism (VTE), transient ischemic attack (TIA), and cerebrovascular accident (CVA) among patients with a history of AF/AFL differ based on CHA_2_DS_2_‐VASc < 2 and >2.

**Study Design**: This retrospective cohort study used the 2016‐2021 National Readmissions Database to identify patients with a diagnosis of AF/AFL on delivery hospitalization encounter. The primary outcome was rates of VTE/TIA/CVA during delivery hospitalization or within 42 days following hospital discharge. Secondary outcomes included rates of severe maternal morbidity (SMM) and cardiovascular‐related SMM. CHA_2_DS_2_‐VASc scores were calculated for each patient at the index hospitalization and scores compared as < 2 and >2. After adjusting for facility characteristics and obesity, weighted logistic regression analyses were used to produce relationships between groups.

**Results**: The cohort consisted of 3,574 delivery hospitalizations with AF/AFL, corresponding to a national estimate of 6,816. Of those, 5,178 (76.0%) had a CHA_2_DS_2_‐VASc < 2 and 1,638 (24.0%) had a CHA_2_DS_2_‐VASc >2. After adjusting for facility and patient characteristics, patients with a CHA_2_DS_2_‐VASc >2 were more likely to experience an acute VTE/TIA/CVA (3.9% vs 1.5%, aOR 2.09 [1.17, 3.73]), SMM (23.5% vs 11.4%, aOR 2.00 [1.59, 2.51]), and pulmonary edema or acute heart failure (11.4% vs 1.3%, aOR 7.56 [4.85, 11.78]). No association was observed between CHA_2_DS_2_‐VASc score and conversion of cardiac rhythm or shock.

**Conclusion**: Pregnant patients with a history of AF/AFL and a CHA_2_DS_2_‐VASc score >2 are significantly more likely to experience a VTE/TIA/CVA following delivery than those with a CHA_2_DS_2_‐VASc < 2. CHA_2_DS_2_‐VASc score at delivery should be considered when determining appropriate candidates for postpartum VTE prophylaxis.

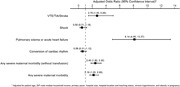

## 97 | A Technology Enabled Solution for Perinatal Mental Health Collaborative Care Models: A Randomized Controlled Trial

Emily S. Miller^1^; David Mohr^2^; Dinah Williams^3^; Melissa Shikany^2^; Tracy Walsh^2^; Nathan W. Winquist^2^; Zara Mir^2^; Elizabeth L. Gray^2^; Shannon R. Smith^2^; Charles Krause^2^; Lara M. Baez^2^; Madhu C. Reddy^4^

*^1^Women & Infants Hospital of Rhode Island and Alpert Medical School of Brown University, Providence, RI; ^2^Northwestern University Feinberg School of Medicine, Chicago, IL; ^3^Women and Infants Hospital of Rhode Island, Providence, RI; ^4^University of California, Irvine, Irvine, CA*

8:30 AM ‐ 8:45 AM

**Objective**: Myriad barriers obfuscate optimal care for perinatal mood and anxiety disorders (PMADs). Collaborative care models (CCMs) integrate mental health into primary care. While CCM offer a promising approach to improving PMAD care, implementation challenges persist, suggesting a need for innovative solutions such as technology‐enabled services (TES) to optimize care delivery.

**Study Design**: Randomized controlled trial (RCT) comparing the impact of a TES to treatment as usual within a CCM on PMAD symptoms. The TES included a cognitive behavioral therapy skills‐based mobile app adapted to the perinatal context with adjunctive SMS‐based coaching. Individuals were eligible to participate in the RCT if they were pregnant or within 3 months postpartum, experiencing ongoing symptoms of PMADs, enrolled in a CCM, and owned a smart phone. The primary and principal secondary outcomes were depression (PHQ9) and anxiety (GAD7) symptoms, respectively, measured serially over the 12‐week intervention. PMAD symptom trajectories were analyzed via generalized linear mixed modeling and as symptom response (50% reduction in symptoms from baseline) and remission (PHQ9 and GAD7 < 4). Satisfaction and engagement with the TES were measured using the Satisfaction Index‐Mental Health and TWente Engagement with Ehealth Technologies Scale, respectively.

**Results**: Of the 75 women enrolled, 38 were randomized to the TES. Depression [β = ‐0.24 (95% CI ‐0.45, ‐0.03)] and anxiety [β = ‐0.24 (95% CI ‐0.46, ‐0.03)] symptom improvement were identified in the total study sample, but no differences were observed between groups in depression [β = ‐0.06 (95% CI ‐0.06, 0.17)] or anxiety [β = ‐0.06 (95% CI ‐0.30, 0.19)] symptoms (Figure 1). Response and remission analyses identified no differences across groups (Table 1). Satisfaction was high but not different between groups whereas participant engagement was higher in the TES group at the mid‐point evaluation.

**Conclusion**: Utilization of a TES to support CCM implementation led to increased engagement in perinatal mental health care but did not result in significant improvements in PMAD symptoms.

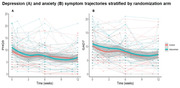

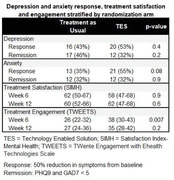

## 98 | Risk of Severe Maternal Morbidity Among Pregnant People with Colorectal Cancer using a Population Database

Shriddha Nayak^1^; Kristin C. Darwin^2^; Arthur J. Vaught^2^; Marika Toscano^1^

*^1^Johns Hopkins University, School of Medicine, Baltimore, MD; ^2^Johns Hopkins University, Baltimore, MD*

8:45 AM ‐ 9:00 AM

**Objective**: Colorectal cancer is among the top three leading causes of new cancer diagnosis as well as cancer death for females in the United States. The purpose of this study was to determine the odds of severe maternal morbidity (SMM) in patients with colorectal cancer in pregnancy using TriNetX, a research network containing real‐time data from 93 health care organizations and > 131 million patients.

**Study Design**: This retrospective cohort study queried TriNetX from inception to 8/2024 for subjects 12‐55 years with a pregnancy diagnosis. Two cohorts were derived: (1) individuals with an active diagnosis of colorectal cancer 1 year prior to, or up to 1 month after, first instance of pregnancy and (2) those without a history of any cancer type in pregnancy. Cohorts were propensity score matched 1:1 by demographics and comorbidities by TriNetX built‐in analysis tools. SMM was defined as CDC's 21 indicators and corresponding ICD‐10 codes. The primary outcome was odds ratio (OR) between cohorts of composite SMM occurring up to 1 year after first instance of pregnancy. Secondary outcomes were odds of individual indicators of SMM. All statistical analyses were conducted in the TriNetX platform.

**Results**: After propensity score matching, 7570 individuals were included in each cohort (Table). Subjects with colorectal cancer in pregnancy had significantly higher odds of composite SMM with OR 4.43 (95% confidence interval (CI) 3.77‐5.22, p< 0.001) compared to those without cancer history in pregnancy. The odds of disseminated intravascular coagulation (DIC) were highest (OR 4.29, 95% CI 3.38‐5.43), followed by acute respiratory distress syndrome (ARDS) (OR 3.73, 95% CI 3.12‐4.46) and hysterectomy (OR 3.03, 95% CI 1.66‐5.56).

**Conclusion**: Patients with colorectal cancer in pregnancy have more than four‐fold increased odds of composite SMM compared to those without cancer history, with considerably higher risk of DIC, ARDS and hysterectomy.

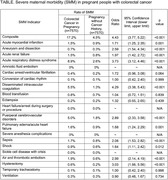

## 99 | Impact of Breastfeeding on Estimated Risk of Postpartum Cardiovascular Disease

Christine P. Field^1^; William A. Grobman^1^; Jiqiang Wu^1^; Anna Palatnik^2^; Mark B. Landon^1^; Denise Scholtens^3^; William Lowe^3^; Nilay S. Shah^4^; Jami Josefson^3^; Sadiya S. Khan^5^; Kartik K. Venkatesh^1^

*^1^The Ohio State University, Columbus, OH; ^2^Medical College of Wisconsin, Milwaukee, WI; ^3^Northwestern University, Northwestern University/Chicago, IL; ^4^Northwestern University, Chicago, IL; ^5^Northwestern University Feinberg School of Medicine, Chicago, IL*

9:00 AM ‐ 9:15 AM

**Objective**: To determine whether breastfeeding is associated with the estimated risk of long‐term atherosclerotic cardiovascular disease (ASCVD) as assessed 10‐14 years after delivery.

**Study Design**: This is a secondary analysis of the prospective Hyperglycemia and Adverse Pregnancy Outcome Follow‐up Study (HAPO FUS). The exposure was any breastfeeding (yes/no). The primary outcomes were estimated ASCVD risk (composite of fatal and non‐fatal coronary heart disease and stroke) over the subsequent 10‐ and 30‐year time periods measured separately. The Framingham Risk Score was used to calculate ASCVD risk 10‐14 years after delivery. ASCVD risk was calculated as a continuous percentage. Multivariable linear regression models were used and adjusted for baseline covariates: field center, age, BMI, height, smoking and alcohol use, parity, and time from delivery to ASCVD risk assessment. Secondarily, we examined whether the association between breastfeeding and ASCVD varied by gestational diabetes mellitus (GDM) status (effect modification).

**Results**: Of 4,540 assessed individuals, the median age was 30.6 years at baseline. Over three‐fourths (79.7%) of individuals reported breastfeeding in this multi‐site international cohort, which did not vary by GDM status (79.5% vs 81.0%). At 10‐14 years after delivery (median 11.6 years), individuals who had breastfed had a lower estimated risk of ASCVD over the subsequent 10 years (adj. Beta: ‐0.13; 95% CI: ‐0.25, ‐0.02) as well as over the subsequent 30 years (adj. Beta: ‐0.36; 95% CI: ‐0.66, ‐0.05). The association between breastfeeding and estimated ASCVD risk varied significantly by GDM status (interaction p< 0.01): the protective effect of breastfeeding for estimated ASCVD risk was over 5‐fold greater for individuals with GDM (TABLE).

**Conclusion**: Individuals who breastfed‐and particularly those with prior GDM‐had a lower estimated risk of long‐term ASCVD. These findings suggest the beneficial impact of breastfeeding for long‐term maternal cardiovascular health, especially among those with GDM.

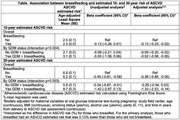

## 100 | Intravenous Ferumoxytol versus Oral Ferrous Sulfate for Iron‐Deficiency Anemia in Pregnancy: A Randomized Controlled Trial

Irogue I. Igbinosa; Stephanie A. Leonard; Ijeoma Iweakogwu; Elizabeth B. Sherwin; Caroline Berube; Deirdre J. Lyell

*Stanford University, Palo Alto, CA*

9:15 AM ‐ 9:30 AM

**Objective**: Iron deficiency anemia (IDA) affects 1 in 10 pregnancies in the U.S. and contributes to maternal and neonatal morbidity. However, the most effective route for iron supplementation in pregnancy is debated. Our objective was to assess the effectiveness of intravenous (IV) ferumoxytol vs. oral ferrous sulfate on hemoglobin (Hb) for the treatment of IDA in pregnancy.

**Study Design**: We conducted a randomized controlled trial of  IV ferumoxytol vs. oral ferrous sulfate.  Pregnant women 24‐34 weeks’ gestation with IDA (ferritin < 30ng/dL or transferrin saturation < 20%, and Hb < 11g/dL) were eligible; hereditary anemias or malabsorptive disorders were excluded. Patients were computer block randomized: 1) Hb 9‐10.9 g/dL received either Ferumoxytol (510mg) or ferrous sulfate (325mg); 2) Hb 7‐8.9g/dL received  Ferumoxytol (1020mg) or ferrous sulfate (650mg). The primary outcome was change in Hb from enrollment to week 4. We estimated that 80 patients were needed, assuming an effect size of 0.5 g/dL, 90% power, 5% type I error rate, and loss to follow‐up rate of 20%. Secondary outcomes included change in Hb by 8 weeks, anemia resolution, and postpartum Hb. Intention‐to‐treat (ITT) analyses were conducted using Wilcoxon rank sum and Fisher's exact tests.

**Results**: Of the 80 enrolled patients, 40 were randomized to oral ferrous sulfate and 40 to IV ferumoxytol. Except for the distribution of BMI categories, baseline characteristics were similar between groups (Table 1). Treatment with IV ferumoxytol resulted in a greater change in Hb at a 4‐week follow‐up than oral ferrous sulfate (median 1.05 (Q1‐Q3 0.68‐1.70) vs 0.40 (Q1‐Q3 0.10‐0.80), p< 0.001) (Table 2). The IV ferumoxytol group also had greater change at 8 weeks (median 1.80 (Q1‐Q3 1.23‐2.48) vs 0.70 (Q1‐Q3 0.20‐1.28), p< 0.001), greater anemia resolution (37/40 (93%) vs. 26/40 (65%), p = 0.005), and postpartum Hb (median 10.25 (Q1‐Q3 9.28‐10.90) vs 9.65 (Q1‐Q3 8.85‐10.05), p = 0.013) (Table 2).

**Conclusion**: Treatment of IDA with IV ferumoxytol improved hemoglobin at delivery and reduced the prevalence of anemia to a greater extent than oral ferrous sulfate.

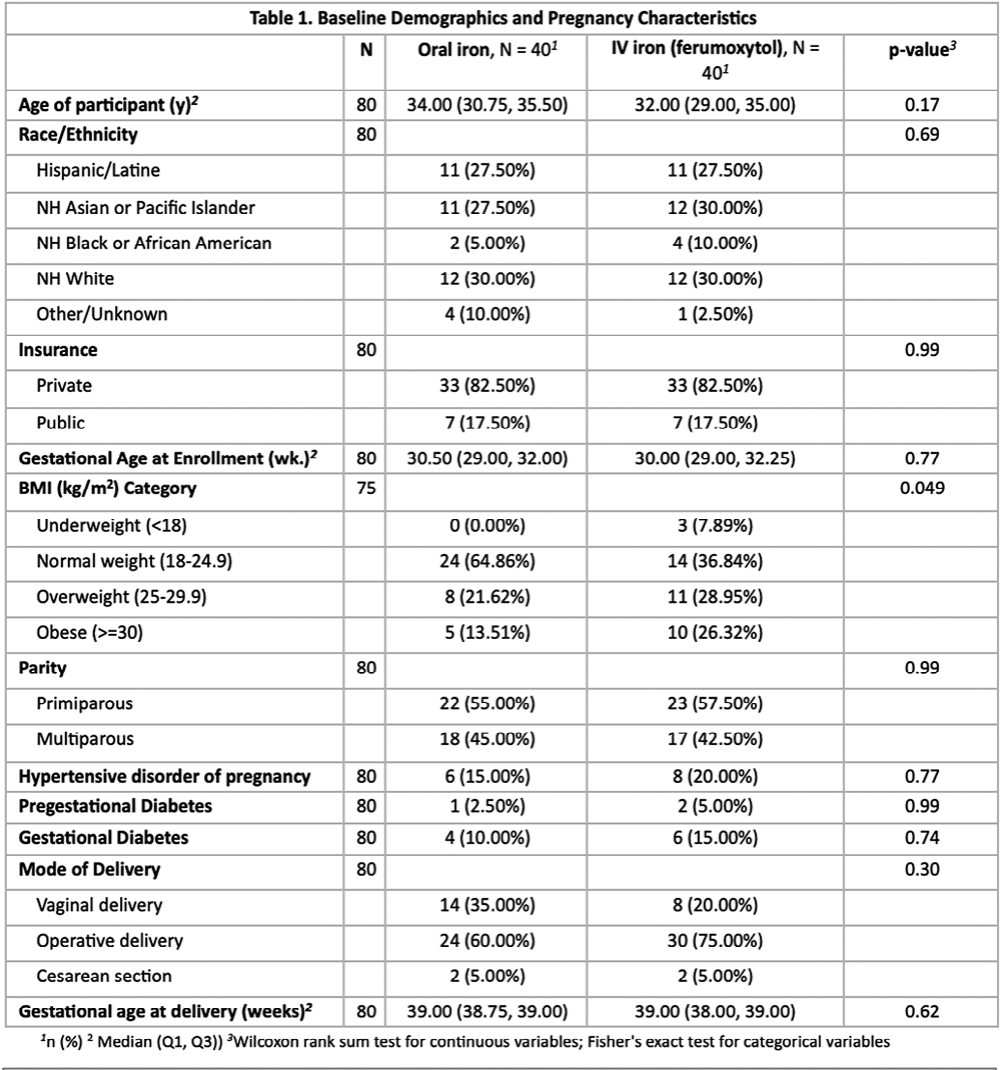

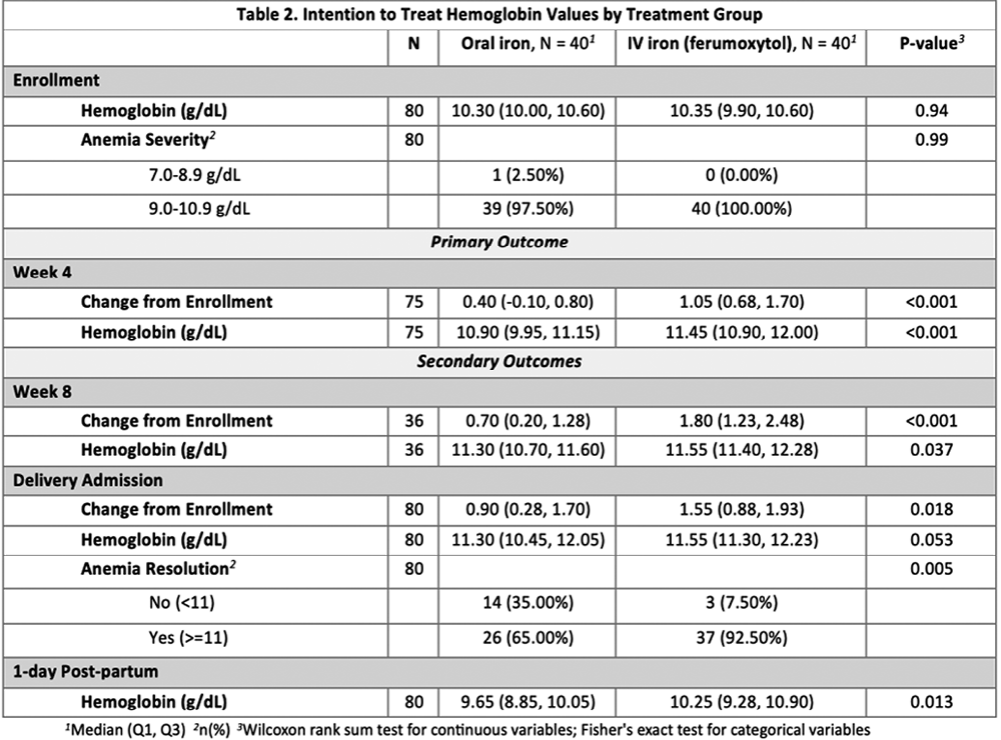

## 101 | Neurodevelopmental Outcomes of 3‐year‐old Children of Mothers with SARS‐CoV‐2 Infection in Pregnancy

Lydia L. Shook; Victor Castro; Laura Ibanez‐Pintor; Roy H. Perlis; Andrea G. Edlow

*Massachusetts General Hospital, Boston, MA*

9:30 AM ‐ 9:45 AM

**Objective**: Large epidemiologic studies have demonstrated an increased risk for neurodevelopmental diagnoses (ND) after maternal viral infection in pregnancy. We previously reported an increased risk of ND in offspring exposed to SARS‐CoV‐2 *in utero* up to 18 months after birth. We sought to determine whether increased risk of offspring ND after maternal SARS‐CoV‐2 infection persisted at 3 years of age.

**Study Design**: Retrospective cohort study of live offspring of all mothers who delivered between March 1, 2020 and May 31, 2021 at 8 hospitals across 2 health systems in Massachusetts. The exposure of interest is a positive SARS‐CoV‐2 PCR during pregnancy. The primary outcome is electronic health record documentation of one or more ICD‐10 diagnostic codes corresponding to ND in offspring. Linear regression models were constructed adjusting for race/ethnicity, insurance status, hospital type, maternal age, and preterm birth.

**Results**: The cohort included 18,124 live births including 861 (4.8%) with maternal SARS‐CoV‐2 in pregnancy‐Table 1. In regression models, maternal SARS‐CoV‐2 was associated with a statistically significant elevation in risk for offspring ND at 3 years (139/861 [16.1%] exposed vs 1653/17,263 [9.6%] unexposed); adjusted odds ratio (aOR) 1.30 [95% CI 1.06‐1.58]; P  =  .01 ‐ Figure 1. The most common NDs were speech/language and motor function disorders. In sex‐stratified models, effects were larger in males (aOR 1.38 [1.06‐1.77]; p = .01) compared to females (aOR 1.20 [0.86‐1.64]; p = .3). Effect was larger in 3rd‐trimester exposure (aOR 1.36 [1.06‐1.71]; p = .01) vs 1st and 2nd trimester exposures (aOR 1.15 [0.83‐1.58]; p = .38).

**Conclusion**: In this large cohort, maternal SARS‐CoV‐2 infection was associated with significantly increased risk for ND among 3‐year‐old children, with a stronger impact on male compared to female offspring and greater effects noted after 3rd‐trimester exposure. Larger cohorts and longer‐term follow‐up with more ND accrued will be required to reliably estimate risk and examine impact of trimester of infection and prior vaccination.

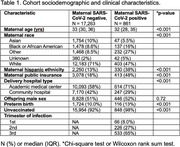

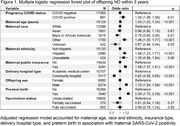

## 102 | Maternal Gestational Weight Gain in GLP‐1 Agonist Exposed and Unexposed Patients

Nishita Pondugula^1^; Jennifer F. Culhane^1^; Lisbet S. Lundsberg^1^; Caitlin Partridge^1^; Audrey A. Merriam^2^

*^1^Yale School of Medicine, New Haven, CT; ^2^Yale New Haven Hospital, New Haven, CT*

9:45 AM ‐ 10:00 AM

**Objective**: To understand the effect of GLP‐1 agonist use in the year prior to conception on gestational weight gain (GWG).

**Study Design**: All patients delivering between 2014‐2024 with evidence of GLP‐1 exposure in the year prior to conception were identified through electronic medical record (EMR) query with hand review to confirm exposure.  GLP‐1 exposed patients were classified by indication for GLP‐1: 1) pregestational diabetes mellitus (PGDM) or 2) weight management (WM) for increased body mass index (BMI). Control groups for each indication cohort were identified from 2021‐2022 delivery EMR data. PGDM controls were patients with pregestational DM1 or DM2 managed with medication other than GLP‐1 agonist in the year prior to conception. WM controls were a stratified random sample of patients without PGDM not on antihyperglycemic medication in the year prior to conception with BMIs between 30 < 40 kg/m^2^ (n = 100) and ≥40 kg/m^2^ (n = 100). Demographic and clinical characteristics and obstetrical outcomes were compared. Logistic regression was employed to assess the crude (OR) and adjusted odds ratios (aOR) for GWG controlling for covariates significant in bivariate tests at < 0.05.

**Results**: 246 patients had GLP‐1 exposure in the year prior to conception. Of these, 104 were exposed for PGDM and 142 for WM. 175 PGDM controls were identified from the two years of EMR data interrogated. Random sampling yielded 200 WM controls.

Bivariate tests for race/ethnicity, pre‐pregnancy BMI, and chronic hypertension identified significant differences between exposed and unexposed patients in the PGDM cohort. For the WM cohort,  exposed and unexposed patients had significant differences in PCOS diagnosis, gestational weight gain, and HDP. Within the PGDM group, GLP‐1 agonist exposure did not significantly affect GWG (Table 2). Those exposed to a GLP‐1 in the WM cohort had significantly decreased risk of GWG below recommendations (aOR = 1.32 (0.71‐2.48)).

**Conclusion**: GLP‐1 agonist use in the year prior to conception decreases risk of GWG under recommendations, which may reflect rebound weight gain during pregnancy after GLP‐1 cessation.

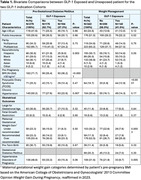

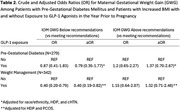

## 103 | Placental Pathology Findings by Timing and Amount of Maternal Cannabis Exposure

Shilpa S. Tummala^1^; Amanda A. Allshouse^2^; Gwendolyn A. McMillin^1^; Jessica M. Comstock^2^; Elizabeth S. Doughty^2^; Robert M. Silver^2^; Torri D. Metz^2^

*^1^University of Utah Health, Salt Lake City, UT; ^2^University of Utah, Salt Lake City, UT*

10:00 AM ‐ 10:15 AM

**Objective**: We aimed to evaluate the association between the timing and amount of maternal cannabis use and placental abnormalities associated with insufficiency.

**Study Design**: We conducted a secondary analysis of individuals enrolled in the nuMoM2b study. Maternal cannabis use was identified by urine immunoassay for delta‐9‐tetrahydrocannabinol (THC‐COOH) and confirmed by liquid chromatography‐mass spectrometry. Exposure was categorized as only 1^st^ trimester or ongoing into 2^nd^ or 3^rd^ trimester. In a secondary analysis, cumulative exposure was calculated using quantitative THC‐COOH values across serial urine samples. Outcomes for above‐median and below‐median exposure were compared. The primary outcome was a composite of maternal vascular malperfusion from placental pathology, including retroplacental clot or hematoma, infarction, accelerated villus maturation, syncytial knots, and decidual hemorrhage. Secondary outcomes were fetoplacental weight ratio, fetal vascularization, or chorioamnionitis. Multivariable modeling adjusted for maternal age, hypertension, diabetes, and nicotine use.

**Results**: Placental pathology was available for 179 participants who used cannabis. Of these, 73 (41%) had only 1^st^‐trimester exposure, and the rest had ongoing exposure. Maternal cannabis use in late pregnancy compared with early was not associated with pathologic findings of maternal vascular malperfusion (Table). The median cumulative exposure to THC‐COOH across pregnancy was 332 ng/mL (Figure). Above‐median cumulative exposure was not associated with maternal malperfusion (Table). There were no differences in secondary outcomes.

**Conclusion**: Maternal cannabis use in late pregnancy was not associated with changes in placental vascularization suggestive of insufficiency when compared with 1^st^‐trimester use alone. Differences in cumulative exposure to cannabis during pregnancy may not correlate with abnormalities in placental vascularization. Further studies are needed to understand the pathophysiology underlying maternal cannabis exposure, placental pathology, and fetal growth restriction.

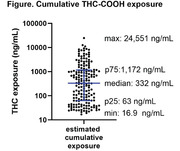

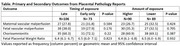

## 104 | The Impact of a Facilitated Postpartum‐to‐Primary Care Transition on Care Utilization within One Year Postpartum

Arlin Delgado^1^; Pichliya Liang^1^; Tierra Bender^1^; Kaitlyn E. James^1^; Alaka Ray^1^; Ishani Ganguli^2^; Jessica L. Cohen^3^; Mark A. Clapp^1^

*^1^Massachusetts General Hospital, Boston, MA; ^2^Brigham and Women's Hospital, Boston, MA; ^3^Harvard T.H. Chan School of Public Health, Boston, MA*

10:15 AM ‐ 10:30 AM

**Objective**: Postpartum to primary care transitions are important for individuals with ongoing care needs after pregnancy, but there are many barriers to effective transitions in practice. This study's objective was to assess if an intervention, which increased PCP visits within four months postpartum by 2‐fold, had a sustained impact on PCP visits and reduced unscheduled care utilization in the first year postpartum.

**Study Design**: This was a planned secondary analysis of an RCT of a behavior economics‐informed intervention, including default visit scheduling, nudge reminders, and tailored messaging, designed to increase PCP engagement within 4 months postpartum versus standard care. The primary outcome in this secondary analysis was receiving an “annual exam” by a PCP within 1 year postpartum. Other outcomes included visits for any reason with a PCP, urgent care visits, and ED visits. Outcomes were compared between the groups with chi‐square testing. Odds ratios with and without adjustment for original randomization strata with robust standard errors were reported.

**Results**: 360 patients were randomized in the original trial (176 control, 184 intervention). 75.4% had anxiety/depression, 19.5% had preexisting/gestational diabetes, and 16.1% had chronic/pregnancy‐related hypertension. Figure 1 shows the outcome comparisons between the two groups. Table 1 lists the unadjusted and adjusted odds and demonstrates that the intervention increased all measures of PCP visits within the first year postpartum, including an increased odds of 2.78 (95% CI 1.75, 4.40) for receipt of an annual exam by a PCP.

**Conclusion**: An intervention designed to facilitate the postpartum‐to‐primary care transition among individuals with chronic conditions resulted in higher rates of PCP visits within the first year after delivery compared with standard care. There were no differences in urgent care or ED visits, though the comparisons may be underpowered. This relatively low‐resource intervention may be an effective strategy for transitioning postpartum individuals’ care back to their PCP.